# Characterization of Ciprofloxacin-Bismuth-Loaded Antibacterial Wound Dressing

**DOI:** 10.3390/molecules25215096

**Published:** 2020-11-03

**Authors:** Dorota Kowalczuk, Małgorzata Miazga-Karska, Agata Gładysz, Paweł Warda, Agnieszka Barańska, Bartłomiej Drop

**Affiliations:** 1 Department of Medicinal Chemistry, Faculty of Pharmacy, Medical University of Lublin, 20-090 Lublin, Poland; agata.gladysz@umlub.pl (A.G.); chemia.lekow@umlub.pl (P.W.); 2 Department of Biochemistry and Biotechnology, Faculty of Pharmacy, Medical University of Lublin, 20-090 Lublin, Poland; malgorzata.miazga-karska@umlub.pl; 3 Department of Informatics and Medical Statistics, Medical University of Lublin, 20-090 Lublin, Poland; agnieszkabaranska@umlub.pl (A.B.); bartlomiej.drop@umlub.pl (B.D.)

**Keywords:** ciprofloxacin-bismuth complex, antimicrobial dressing, antimicrobial delivery, wound dressing, wound infection

## Abstract

The research was focused on developing a potentially antibacterial wound dressing made of polyurethane foam and loaded with bismuth-ciprofloxacin (Cip-Bi). The Cip-Bi chemical structure was confirmed by Fourier transform infrared spectroscopic (FTIR) analysis. The sought after antibacterial wound dressing was obtained by modification of the raw dressing with an iodine or bromine solution and subsequently with a Cip-Bi hydrogel. The amount of Cip-Bi loaded into the dressing matrix was determined indirectly on the basis of the differences in Cip-Bi concentrations, before and after the modification process, and the determination was performed with the HPLC (high-performance liquid chromatography) method. The modified dressing was found to have a two-step release of Cip-Bi, a feature helpful in the treatment of locally infected wounds and prevention of secondary bacterial infection. The zone of inhibition test against the selected Gram-positive and Gram-negative bacteria confirmed the antibacterial activity of the Cip-Bi-modified dressing. Preliminary tests conducted so far have been indicative of the Cip-Bi dressing’s relatively high activity against the tested organisms.

## 1. Introduction

Fluoroquinolones are representatives of an important group of chemotherapeutic compounds with a broad spectrum of activity against various pathogenic microorganisms resistant to aminoglycosides, penicillins, cephalosporins, tetracyclines and other antibiotics. In general, they exhibit activity against aerobic Gram-negative and Gram-positive bacteria, while they are especially active against the aminoglycoside-resistant Pseudomonas aeruginosa and beta-lactamase-producing organisms [1]. Their mechanism of action relies on inhibition of topoisomerase II (DNA gyrase) and topoisomerase IV, both of which are substantial for DNA replication [2]. It has been suggested that metal complex intermediates participate in this process [3] in such a way that in the presence of metal ions, the uptake of quinolones by the bacterial cells is higher in comparison with the drugs acting alone. Thus, it is plausible that the formation of metal complexes increases the bioavailability of metal ions or ligands, or both [4].

Combining metals with pharmaceutical agents can provide a strategy to improve drugs’ activity, decrease their toxicity and overcome resistance to their action. Quinolones, due to their ability to coordinate metal ions, may find a particularly interesting application in this strategy [5]. Therefore, it is not surprising that a number of studies on the issue of interaction between fluoroquinolones and metallic ions are found in the specialist literature. Abd El-Hamid, S.M. et al. [6] reported synthesizing Zn(II), Zr(IV), Ce(III), Th(IV) and U(VI) complexes containing ofloxacin and 2,2′-bipyridine. They described the inhibitory effect of these complexes on Gram-positive and Gram-negative bacteria, fungi and in vitro anticancer activity against breast (MFC7) and colon (HCT-116) cells as a potentially antibacterial, antifungal and antitumor activity of these agents. The subject of platinum(II) complexes with antitubercular activity was also raised by de Oliveira et al. [7]. Furthermore, Kostelidou A. et al. [8] reported promising antibacterial properties of the getifloxacin metal complexes with copper(II).

Ciprofloxacin (1-cyclopropyl-6-fluoro-4-oxo-7-piperazin-1-ylquinoline-3-carboxylic acid) is a member of the second generation quinolone antibiotics family and it can be used in the treatment of complicated and uncomplicated urinary tract infections, lower respiratory tract infections, skin and skin-structure infections, urethral and cervical gonococcal infections, bone and joint infections, infectious diarrhea, typhoid fever and acute sinusitis [9]. Due to its specific biological and chemical properties, ciprofloxacin has also found application in preparation of metal complexes [10,11,12,13].

This paper presents the process of developing a new, wound healing, antibacterial dressing loaded with a ciprofloxacin-bismuth complex and its characterization, performed with the use of several analytical and microbiological methods. All this has resulted in producing a new type of wound dressing, composed of a hybrid material, which is capable of a gradual release of the ciprofloxacin-bismuth(III) complex. This newly obtained dressing exhibits an antibacterial activity both against the reference bacterial strains and clinical isolates from the infected wounds and appears to be more active on the Gram-negative *E. coli (Escherichia coli)* strain than the Gram-positive *S. aureus (Staphylococcus aureus)* strain.

## 2. Results and Discussion

**Scheme 1:**
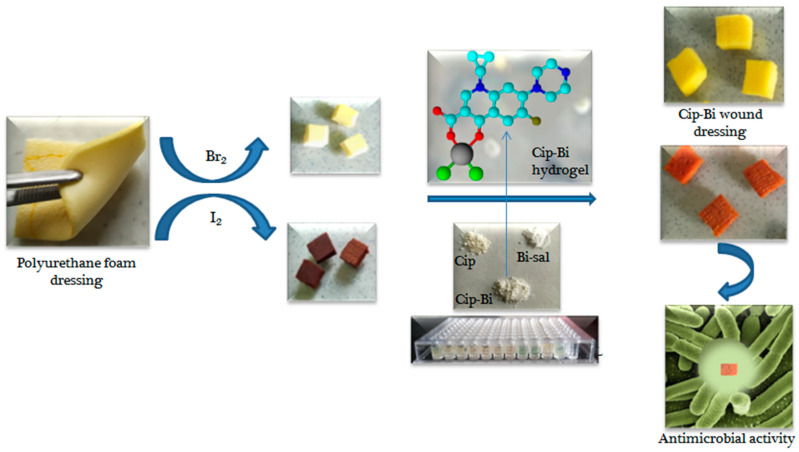
Synthesis and activity of ciprofloxacin-bismuth-loaded dressing.

Chitosan and its derivatives have been widely used in various fields of cosmetics and medicine including tissue engineering and wound dressings. Chitosan-based hydrogels could provide a moist wound environment, a protective role in secondary infections, remove wound exudate, and promote tissue regeneration and faster wound healing [14]. Chitosan-based hydrogel has been used primarily for its wound healing, regenerating and antibacterial properties. Antibacterial properties of chitosan hydrogel were intensified by loading Cip-Bi complex. Thus, the foam dressings selected from the commercially available ones were subjected to modification with the use of hydrogel composed of chitosan crosslinked with the glutaraldehyde and Cip-Bi complex, and subsequently evaluated with respect to the drug load, drug releasing profile in vitro and antibacterial activity (Scheme 1). 

For modification, foam material was used with a porous structure having the ability of solution absorption into open, narrow spaces by capillary action. Polyurethane foams are one of the most commonly used wound dressings. Polyurethane foam dressings belong to dressings that exhibit the ability to absorb exudates and provide a moist wound environment. By exudate management together with a better control of the pH and the oxygen permeability [15], these type of dressings have potential to promote faster healing, tissue re-epithelialization and prevent the formation of scarring. Besides, the foams are easy to remove, protect the skin around the wound, maintain temperature, provide mechanical protection, are nontoxic and nonallergenic and are used in the healing of acute and chronic wounds [15].

Bioactive dressing materials contain functional groups that can react with other molecules via hydrogen bonds and electrostatic interactions. As the studies have shown, the modification of the wound dressing leads to a decreased activity of proteases, such as metalloproteinases and elastase, in an attempt to stimulate wound healing [16].

The wound healing, especially of chronic wounds, may be complicated by bacterial colonization. Generally, the infected wounds are treated with systemic antibiotics. Thus, the use of dressings containing antibacterial agents can be a valuable option to extinguish the infection in the course of wound healing. 

Ciprofloxacin, one of the fluoroquinolone group of antibiotics with a broad spectrum of activity, was used as the antibacterial protection for the dressing. Since metal-drug complexes are potentially capable of showing a greater antimicrobial activity when compared to the uncomplexed ligands, we decided to use the bismuth-ciprofloxacin complex instead of ciprofloxacin alone, surmising that it will raise the bioactivity against the resistant species [17]. We also assumed that due to lower solubility of the Cip-Bi complex in comparison to the parent drug, it will reach a higher concentration in the wound.

As the literature has shown, quinolones represent the second most common antibiotic group associated with allergic reactions, which can be classified into 2 types: Ig-E-mediated immediate reactions and T-cell-mediated delayed reactions. Allergic sensitization to ciprofloxacin occurs relatively often (up to 30% of anaphylaxis and 35% of delayed hypersensitivity) [18]. So, the application of nonabsorbable chelate complex may potentially reduce the body’s allergic response to ciprofloxacin.

It is likely that in a wound, hydrolysis of the complex with the release of bismuth may occur. It is also possible that a compound containing bismuth will treat the wound as well as inhibit inflammatory reactions. Therefore, preliminary skin monitoring tests were carried out following application of the modified samples of the dressings. This was conducted with the use of the skin analysis system with photographic documentation. The results confirming the therapeutic potential of the modified dressings will be shown in the later work.

**Figure 1:**
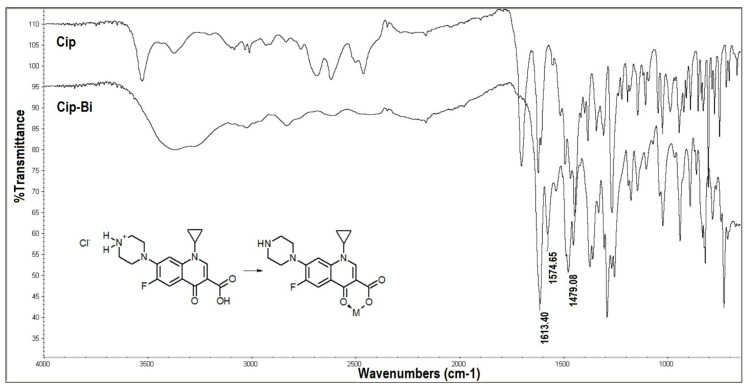
Fourier transform infrared (FTIR) spectra obtained for ciprofloxacin (Cip) as hydrochloride and ciprofloxacin-bismuth complex (Cip-Bi).

The spectrum of Cip (Figure 1) is compatible with the literature data [19]. It includes characteristic absorption bands corresponding to specific vibrations: at 3528 and 3363 cm^−1^ (O-H stretching vibrations of carboxyl group), at 3100–3012 cm^−1^ (C-H stretching vibrations of the aromatic ring), at about 2929 cm^−1^ (C-H symmetrical and asymmetrical stretching vibrations of the methyl and methylene groups), at 2691–2461 cm^−1^ (the region of stretching vibrations of the ionized amine groups) and the intense absorption bands at 1701 cm^−1^ and 1624 cm^−1^ related to the stretching vibrations of carboxy carbonyl (C=O of -COOH) and carbonyl (C=O) in position 4 of the quinoline ring respectively, as well as the absorption bands in the region of 1510–1446 cm^−1^, which correspond to the C=C stretching vibrations of the aromatic ring. The Cip-Bi spectrum compared to the Cip spectrum shows the absence of a peak at 3528 cm^−1^ and a peak at 1701 cm^−1^, and the appearance of peaks at about 1575 and 1479 cm^−1^, due to the symmetrical and asymmetrical carboxylate stretching vibrations (COO-), as well as the peak of out-of-plane bending vibrations (COO-) of medium intensity at about 830 cm^−1^, while the peak of the carbonyl group (4-oxo) has slightly shifted toward the lower frequencies (from 1624 to 1613 cm^−1^). The observed changes provide evidence for the substitution of protons in the carboxylic group of ciprofloxacin (deprotonation) by a Bi-containing cation. Bismuth is chelated to the ciprofloxacin molecule via the Bi-O bond through the carboxylate anion and the O → Bi bond through the 4-oxo group. The obtained spectra are similar to the spectrum reported by Shaikh et al. [20].

**Figure 2:**
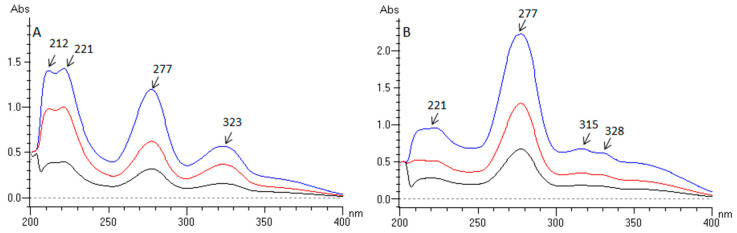
UV absorption spectra of ciprofloxacin (**A**) and ciprofloxacin-Bi complex (**B**) as a function of the concentration in μg/mL: 5 (back line), 10 (red line), 20 (blue line).

The complexation process by influencing the electron transfer in the chromophore caused a modification of the UV (ultraviolet) absorption spectrum of the Cip molecule. The spectral effects associated with a wavelength shifting, peak disappearance and changes in the intensity of the bands characteristic of Cip were observed. The absorption spectrum of Cip in an acidic environment shows a strong absorption band at 277 nm and two much less intense bands at 312 and 328 nm (Figure 2B). Upon complexation with Bi(III), the bands in the range of 310–330 nm were modified. The first peak disappeared, and the second peak was shifted (Figure 2B), with the result that the medium intense peak at 323 nm appeared (Figure 2A). As the literature has reported, the shift of the peak in this region can suggest the formation of a six-member ring as a result of the attachment of Cip to the metal via the carboxylate and ketone groups. As a consequence, such modification can disturb the conjugation system of the Cip structure [21]. Besides, the UV spectrum of the Cip-Bi complex showed a hyperchromic effect in the short-wave region: 221–212 nm (Figure 2A).

**Figure 3:**
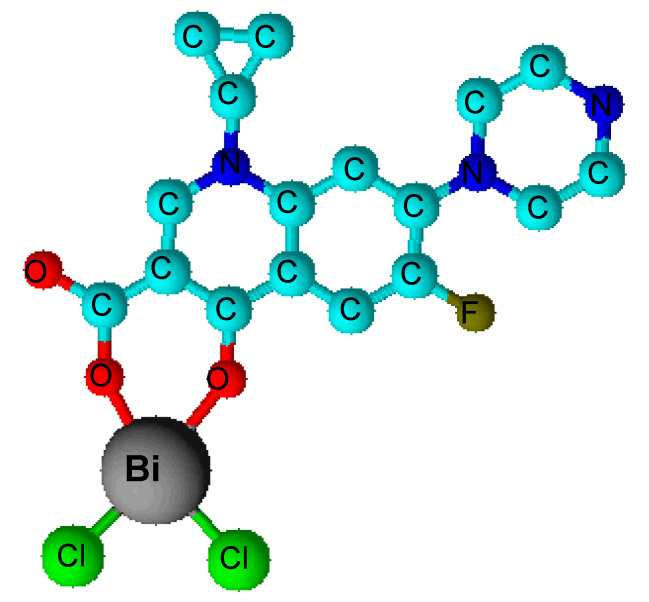
Suggested three-dimensional (3D)-structure for ciprofloxacin-bismuth complex.

The increase in absorbance was proportional to the increase in molar concentration for both Cip and Cip-Bi (at λ = 277 nm: y = 34.69x + 0.02, R = 0.9999 for Cip-Bi and y = 29.85x + 0.20, R = 0.9975 for Cip; ratio of the slopes of regression lines, Cip-Bi/Cip: 1.16). Molar absorptivity was determined to evaluate the number of moles attached to the bismuth atom in the Cip-Bi complex [17]. The molar ratio analysis was based on the comparison of molar absorptivity of the Cip-Bi complex with the molar absorptivity of the Cip ligand. The obtained molar ratio Cip-Bi/Cip was 0.96 at λ = 277 nm. The performed evaluation exhibited the existence of a 1:1 molar ratio of metal to ligand. No significant differences were observed in the absorbance of Cip-Bi (at 277 nm) recorded before and after acidic hydrolysis. A simple qualitative test (Beilstein flame test) confirmed the presence of chlorine in the Cip-Bi compound. Quantification of bismuth by a titration method indicated the content of bismuth at 33.40% ± 2.44% (theoretical value: 34.25%). Figure 3 shows the probable structure of the synthesized Cip-Bi complex. The content of Cip in the presented structure is 54%.

The HPLC method was developed [22] for the quantitative assessment of Cip-Bi incorporated into the dressing matrix and released further on. UV-detection and FL (fluorescence)-detection (for control monitoring and recording low concentration levels) were used for the determination of Cip-Bi analyte and calibration curves. The calibration curve (Mandel’s linearity test: TV (tested value) = 1.65 − 2.32 < F99% (1.15) = 8.68, *n* = 18) was constructed by plotting the peak areas of Cip-Bi standard solutions versus the concentrations in the range of 3.0–18.0 μg/mL with the correlation coefficient (R) greater than 0.9995. A typical linear regression equation for the calibration curve was y = 69,898 ± 412x − 45,270 ± 4808 (R^2^ = 0.9994) for HPLC with UV-detection, or y = 42,926,873 ± 234,546x − 14,396,984 ± 2,740,277 (R^2^ = 0.9995) for HPLC with FL-detection. 

The detection and quantification limits (determined on the basis of the standard deviation of residues of the linear regression curve obtained for a dilution series of 3.0 μg/mL standard solution) were found to be 0.04 and 0.11 μg/mL for FL-HPLC or 0.12 and 0.37 μg/mL for UV-HPLC, respectively. The intraday and interday precision estimated by successive injections of three individual standard samples containing 5, 10 and 15 μg/mL of Cip-Bi were varied in the range of 0.39–2.77% and 2.52–4.46% (relative standard deviation, RSD), respectively.

The HPLC method [22] was satisfactorily applied for determination of the Cip-Bi hydrogel solutions before and after the dressing modification. The chromatographic analysis revealed slight differences in the concentration of both solutions. These observations allow a conclusion that there were no chemical interactions between the Cip-Bi complex and the dressing matrix and the observed changes in the concentration could be a result of the measurement uncertainty. The quantity of Cip-Bi loaded into the dressing matrix was estimated on the basis of the Cip-Bi content in the absorbed hydrogel volume. The obtained results are summarized in Table 1.

**Figure 4:**
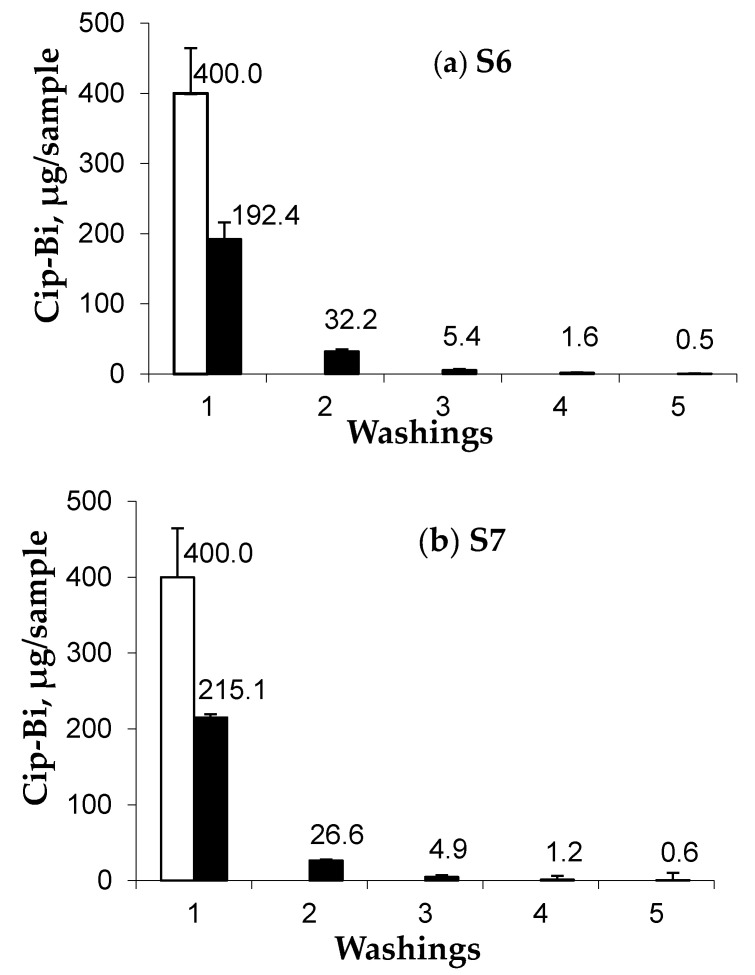
The amount of the ciprofloxacin-bismuth (Cip-Bi) introduced into the dressing sample (colorless bar) and released from the dressing sample (black bar). (**a**) S6—bromine-activated, Cip-Bi hydrogel-loaded dressing; (**b**) S7—iodine-activated, Cip-Bi hydrogel-loaded dressing.

In order to prevent the secondary bacterial infection, an ideal wound dressing should be expected to release an antibacterial agent. Controlled drug release provides localized delivery and minimizes the risk of systemic side effects. Polymer-based dressings such as polyurethane foams and hydrogels are often used for controlled drug delivery to wounds. Drug release from polymeric carriers is mostly caused by hydration of the polymer, swelling, drug diffusion through the matrix or matrix erosion. In practice, most of the wound dressings release the drugs embedded in the matrix by a combined mechanism [23]. The probable release profile of Cip-Bi in vitro from the modified fragments of polyurethane dressing was evaluated by rinsing them with diluted hydrochloric acid and determining the content of Cip-Bi in the washes. In general, a two-step release of Cip-Bi from the dressing sample was observed. In the first period of this process, the drug was released quickly (about 50%), and in the second period, the release was significantly slower. A similar release trend was observed regardless of the dressing modification method. This important increase in the drug dissolution in the initial period could be a result of the hydrogel swelling which increases the dissolution area. The drug release characteristics are illustrated in Figure 4. Initially, a loading dose maximizing killing of microbes and then lower maintenance doses were observed. Such an antimicrobial drug release profile can be useful for the treatment of a locally infected wound as well as prevention of a secondary bacterial infection. A treatment consisting in a high initial dose followed by an extended tapering of doses is found to optimize the use of antibiotics, improve the success of eradicating infections and prevent the appearance of resistance [24].

It was found that the majority of bacteria that cause wound infections are resistant to a minimum of one of the most commonly used antibiotics. Moreover, infectious strains become resistant to almost all classes of antibiotic, which makes it necessary to look for new substitutes [25]. Since the effectiveness of ciprofloxacin-based therapy is known to be compromised by bacterial resistance, new and more powerful drug strategies, which could be able to fight this bacterial resistance, are required [26]. Therefore, we found it necessary to develop new therapies based on an antibiotic-metal hybrid. 

In our study, the samples were tested for activity against selected medically relevant pathogens, including, what is important, clinical strains: Gram-negative (*E. coli*) and Gram-positive (*S. aureus*) bacteria. First, the MICs (minimum inhibitory concentrations) values of the synthesized Cip-Bi complex as well as the parent components, i.e., Bi (bismuth citrate) and Cip (ciprofloxacin hydrochloride), were evaluated. As Table 2 shows, Bi presented a lower antibacterial activity, from 167 to 400 µg/mL, when compared to Cip and the Cip-Bi complex. The MICs of the latter were similar to each other (regardless of bacterial reference or clinical bacterial strains) and they were in the range from 0.0065 to 0.4163 µg/mL. It was found that all the tested compounds, i.e., Bi, Cip and Cip-Bi, showed higher activity in the inhibition of *E. coli* than *S. aureus*. Significant differences in antibacterial activity of these compounds against *E. coli* and *S. aureus* strains were confirmed by Tukey’s test: *p* (0.002–0.03) < 0.05. 

However, it should be noted that the Cip-Bi complex exhibited similar potency as the ligand (Cip) against *S. aureus* strains, while its activity against both the reference and clinical strains of *E. coli* from the infected wounds was much higher (Table 2). 

The complexation with bismuth resulted in an intensification of the activity of ciprofloxacin against *E. coli* bacteria (*p* < 0.05, Tukey’s test).

The antibacterial activity of Cip-Bi-loaded dressing samples was subsequently evaluated using the plate antimicrobial test in the solid medium. The obtained results are presented in Table 3.

**Figure 5:**
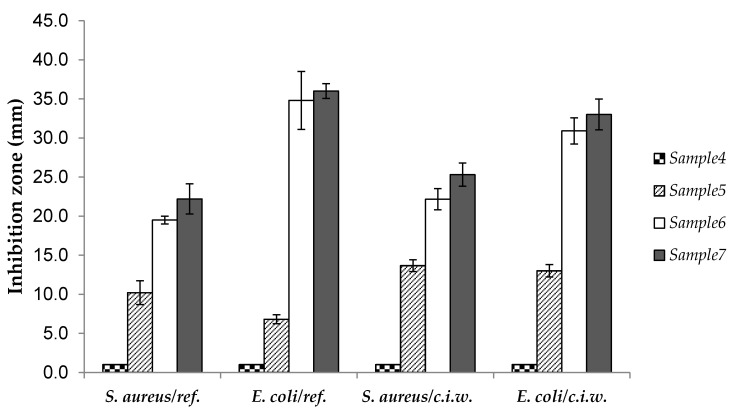
A representative graph comparing the antibacterial activity of the ciprofloxacin-bismuth (Cip-Bi) dressing samples: Sample 4 (bromine-activated, hydrogel-loaded dressing); Sample 5 (iodine-activated, hydrogel-loaded dressing); Sample 6 (bromine-activated, Cip-Bi hydrogel-loaded dressing); Sample 7 (iodine-activated, Cip-Bi hydrogel-loaded dressing) against the tested, reference (ref.) and clinical strains of bacteria (c.i.w.—clinical isolates from infected wounds)**.**

The zones of inhibition, on the Mueller Hinton agar, around the modified dressings containing approximately 0.4 mg of Cip-Bi, were 19.5–36.0 mm. The untreated dressing (sample (S)1), similarly to the dressing activated with bromine (S2) or iodine (S3), showed no inhibition zones against the reference strains. The bromine-activated dressing loaded with hydrogel (S4) showed an insignificant activity, whereas the activity of the dressing with hydrogel but activated with iodine (S5) was slightly higher against both bacterial species (7–10 mm and 13–15 mm for the reference and clinical strains, respectively). However, the antibacterial activity of the Cip-Bi-loaded dressings (S6, S7) was by far the largest, as it is illustrated in Figure 5. In the case of the reference strains, the Cip-Bi-loaded dressings were about twice as active as the iodine-treated dressings against *S. aureus* and about five times more active than the iodine-treated dressing against *E. coli*. 

A significant increase in antibacterial activity for the Cip-Bi hydrogel-loaded dressings (experimental samples: S6 and S7) in relation to the hydrogel-loaded dressing without Cip-Bi complex (control samples: S4 and S5) was confirmed by Tukey’s test (*p* (0.0002–0.02) < 0.05). 

It should be noted that activating the dressings prior to loading the Cip-Bi compound intensified their final antimicrobial activity, especially against *E. coli* compared to the non-activated ones. The zones of growth inhibition around the activated samples ranged from 26 to 36 mm (Table 3), while those zones for non-activated samples were 14–19 mm.

**Figure 6:**
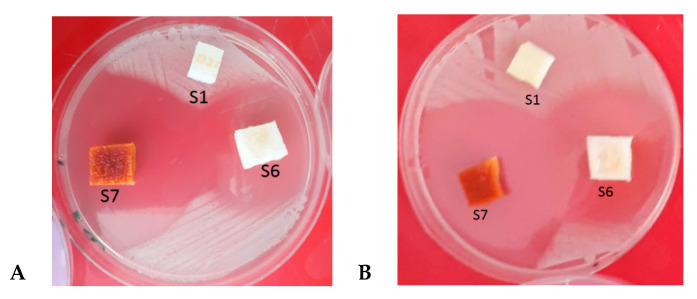
Exemplary photographs showing the antibacterial activity of the tested dressing: S6—modified Br_2_/Cip-Bi hydrogel and S7—modified I_2_/Cip-Bi hydrogel in relation to the untreated dressing (control sample S1) against *S. aureus*_c.i.w_ (**A**) and *E. coli*_c.i.w_ (**B**) strains.

Assessment of the antibacterial efficacy of the newly modified wound dressings against the clinical bacterial strains appears to be pivotal. Our preliminary tests seem to confirm the ability of the Cip-Bi-modified dressings to inhibit the growth of the bacteria isolated from either the patient’s wound or the patient’s urine. The clinical strains presented a slightly higher susceptibility to activating agents, especially towards iodine, compared to the reference strains. The iodine-activated, Cip-Bi-modified dressings (S7) showed the highest inhibitory activity against all the tested clinical strains, while the bromine-activated, Cip-Bi-modified dressings’ activity was slightly lower (S6). It is worth noting that both tested hybrid biomaterials (S6, S7) were effective against the reference as well as clinical strains. However, in the case of clinical strains, similarly to the reference strains, the tested Gram-negative bacteria were less resistant to the activity of the modified dressings than Gram-positive bacteria. Figure 6 illustrates the representative photographs showing the antibacterial activity the Cip-Bi-modified dressings.

The present study confirms an increased antibacterial activity of the Cip-Bi-loaded dressings in comparison to unmodified dressings. Apparently, the CIP-Bi-loaded dressings exhibit a high antimicrobial potential and may effectively protect wounds from infections. 

## 3. Materials and Methods

Pure ciprofloxacin (Cip) as hydrochloride was obtained from Polfarma (Starogard Gdanski, Poland). Chitosan (medium molecular weight, 75–85% deacetylated physical form) and poly(ethylene glycol), PEG-200, were purchased from Sigma-Aldrich Co. (St. Louis, MI, USA). Biatain® non-adhesive polyurethane foam dressing (Coloplast, Warsaw, Poland) was supplied by a local medical supplier. Bismuth(III) citrate (Bi), iodine, bromine, triethylamine, hydrochloric acid (0.1 M), fixanal 1 L Ampoule (from Sigma-Aldrich Co.) and 25% ammonium hydroxide solution (from STANLAB, Lublin, Poland) were also purchased. Methanol and acetonitrile (both of HPLC grade), 35–38% hydrochloric acid, 85% orthophosphoric acid and other reagents and solvents of analytical grade were acquired from Avantor Performance Materials, Gliwice, Poland S.A. The water used in the experiments was double-distilled.

### 3.1. Preparation of the Bismuth-Ciprofloxacin Complex

The bismuth-ciprofloxacin complex was synthesized as a result of the bismuth reaction with ciprofloxacin performed in compliance with the previously described procedure [20] and the author’s modifications. To outline it only briefly, the solution of Cip (as hydrochloride) in distilled water was added drop by drop to the acidic solution of a bismuth salt. The resulting mixture was further basified with an ammonia solution (10%, *v*/*v*) and mixed by constant stirring for about 5 h at 50 °C. The precipitated complex was filtered out and washed with a mixture of methanol-water (1:1 *v*/*v*) until the filtrate was free of Cip in TLC (thin layer chromatography). The obtained product was dried under vacuum.

### 3.2. FTIR Characterization of the Bismuth-Ciprofloxacin Complex

The chemical structure of Cip-Bi was examined with attenuated total reflection–Fourier transform infrared spectroscopy (ATR–FTIR) using a Thermo Scientific Nicolet 6700A spectrometer (Waltham, MA, USA) equipped with a single-bounce beam path, a diamond crystal at a 45° angle of reflection and OMNIC 8.1 computer software. The Cip-Bi with respect to Cip was analyzed over the region from 600 to 4000 cm^−1^ at the spectral resolution of 4 cm^−1^. All spectra were averaged over 32 scans.

### 3.3. UV Characterization of the Bismuth-Ciprofloxacin Complex

The absorption spectral measurements were carried out on a UV-vis Hitachi U-2001 spectrophotometer (Hitachi Instrument Inc., Tokio, Japan) controlled by UV Solutions software using 1 cm matched quartz cells. The solutions of Cip and Cip-Bi in the concentration range of 5–20 μg/mL (0.017–0.069 mM/L for Cip; 0.009–0.034 mM/L for Cip-Bi) were prepared in 5% hydrochloric acid. The ultraviolet spectra were recorded in the range 200–400 nm. 

### 3.4. Titration of Bismuth

An accurately weighed amount of 0.2 g of Cip-Bi compound was dissolved in 10 mL of 10% nitric acid and heated in a boiling water bath for 5 min. Next, 0.1 g of potassium chlorate was added, the solution was heated for another 5 min and 100 mL of a distillate water was added. The solution was titrated with 0.05 M sodium edetate against xylenol orange as an indicator.

### 3.5. Preparation of the Modified Dressing Samples

The samples of the polyurethane foam dressing of about 0.5 × 0.5 cm in size were modified using a two-step approach. Initially, the samples were activated using two activating agents and next, they were loaded with a hydrogel containing ciprofloxacin-bismuth complex (Cip-Bi hydrogel). In order to obtain the Cip-Bi hydrogel, 5 mg of chitosan and 15 mg of bismuth-ciprofloxacin complex were dissolved in 10 mL of 10% citric acid, then 5% glutaraldehyde and PEG-200, each in the amount of 0.3 mL, were added and a mixture was made up to 15 mL with ethanol (Cip-Bi concentration was 1 mg/mL). In the method 1, the samples were gently stirred with a 1% ethanolic iodine solution for 1 h at 37 °C. The excess solvent was removed by placing the samples on the filter paper. Next, the samples were treated with the Cip-Bi hydrogel. In the method 2, the samples were prepared in the same manner, but using a 1% ethanolic bromine solution as an activating agent. The samples subjected to further qualitative tests were previously dried at 37 °C.

### 3.6. HPLC Determination of Loaded and Released Cip-Bi 

Chromatographic analysis was carried out at λ = 279 nm (spectrophotometric (UV) detection) and at λex 279 nm/λem 430 nm (fluorescence (FL) detection) on the Waters, e2695 Aliance, HPLC system (Waters Co., Milford, CT, USA) equipped with the Purospher STAR RP-18, 4 × 125 mm (5 µm), column (Darmstadt, Germany), and the Waters Empower Pro v.2 software for acquiring, processing, reporting and managing the chromatographic information using a mixture of triethylamine–acetonitrile–methanol–phosphoric acid (10 mM) (0.2:25:25:50, *v*/*v*/*v*/*v*) as the mobile phase at a flow rate of 0.7 mL/min.

In order to obtain the Cip-Bi stock solution, 10 mg of the tested substance was dissolved in 5% hydrochloric acid and diluted to the volume of 10 mL with the same acid. Calibration solutions were prepared in triplicate in the concentration range from 3 to 18 µg/mL. In 10 mL volumetric flasks, an appropriate volume of stock solution was made up to the mark with 5% hydrochloric acid. These standard solutions were injected into the chromatographic system. UV-detection and FL-detection (as control monitoring) were used for the determination of the Cip-Bi analyte, and calibration curves were generated on the basis of the obtained data. 

### 3.7. Evaluation of Loaded Cip-Bi

This test was conducted for the foam dressing samples (about 0.5 × 0.5 cm, 20 mg) in order to check how much Cip-Bi was loaded into the samples. The dressing samples activated with iodine or bromine solution were added to the Cip-Bi hydrogel solution (containing 1 mg of Cip-Bi per 1 mL) and gently stirred at 37 °C. The samples of Cip-Bi hydrogel solution, before and after modification of the dressing, were diluted in the 1:100 ratio, filtered through a 0.45 membrane filter and subsequently determined with the use of the UV-FL HPLC method. The concentration of Cip-Bi in the hydrogel solutions was calculated from the calibration equation after multiplying by the dilution factor. The quantity of Cip-Bi hydrogel solution absorbed by the dressing sample was evaluated from the volumes of the Cip-Bi hydrogel solution, before and after dressing modification.

### 3.8. Evaluation of Released Cip-Bi 

The Cip-Bi release study conducted in vitro was to estimate the releasing profile of Cip-Bi from the drug-loaded samples (the polyurethane foam dressing of 0.5 × 0.5 cm in size, modified with Cip-Bi hydrogel, containing 400 μg Cip-Bi compound). The Cip-Bi-loaded samples were immersed in 2 mL of 0.1 mol/L hydrochloric acid and shaken repeatedly every 30 min with a fresh portion of the washing solution at 37 °C. The quantity of Cip-Bi in the washing solutions was determined directly by the UV-FL HPLC method and calculated from the calibration equation.

### 3.9. Evaluation of the Antimicrobial Activity

The zones of bacterial growth inhibition caused by the tested samples, previously modified in compliance with the methods 1 and 2 (the polyurethane foam dressing of 0.5 × 0.5 cm in size, modified with Cip-Bi hydrogel, containing 400 μg Cip-Bi compound), were evaluated for the reference microorganisms from the American Type Culture Collection (ATCC), including Gram-negative bacterium (*Escherichia coli* ATCC 25992) and Gram-positive bacterium (*Staphylococcus aureus* ATCC 25923). The clinical bacterial isolated (*Escherichia coli, Staphylococcus aureus*) from infected wounds or urine were obtained from the University Hospital patients and stored in the strain bank at the Department of Biochemistry and Biotechnology, the Medical University of Lublin, Poland. The stock cultures were maintained at −70 °C in the Tripticase Soy broth with 16% (*v*/*v*) glycerol until the study was performed. Before the experiments, each bacterial strain was passaged onto a fresh Mueller-Hinton agar (M-H) (Oxoid, UK) at 30 °C for 48 h. Next, the inocula were prepared with fresh microbial cultures in sterile 0.9% NaCl to match the turbidity of 0.5 McFarland standard. The antibacterial activity of the samples against the tested bacteria was evaluated by measuring the zones of inhibition in the disk diffusion method (Kirby-Bauer Disk Diffusion Susceptibility Test Protocol). Antibacterial disc diffusion assays were carried out on Petri plates with solid medium (M-H agar). Appropriate strain cultures were separately spread over the agar surface using a cotton swab. Next, the tested liquid samples, i.e., Bi in 10% hydrochloric acid (10 μL of 2.0 mg/mL solution), Cip and Cip-Bi in 10% citric acid (10 μL of 2.0 mg/mL solution), or previously prepared pieces of the modified dressing with about 0.4 mg of active substance, were placed on a sterile disc or directly on the Petri plates containing the agar medium. After 18 h of incubation at 37 °C, the zones of microbial growth produced around the tested samples were measured and recorded as the diameters of inhibition (mm). The unmodified material polyurethane foam dressing (Coloplast) was tested as a negative control (S1), while the dressing activated with a bromine solution (S2) or iodine solution (S3), both loaded with the hydrogel without Cip-Bi complex (S4 and S5, respectively), were used as positive controls in the microbial tests.

In order to obtain final concentrations of the tested samples, ranging from 0.00061 to 40 µg/mL (in case of Cip and Cip-Bi) or from 0.00611 to 400 µg/mL for Bi, the Minimal Inhibitory Concentration (MIC) value was determined (against the CLSI criteria, Clinical and Laboratory Standards Institute (CLSI), 2018) with the M-H bullion (Oxoid, UK), using their serial two-fold dilutions. As the control background, the antibacterial activity of the used solvents was evaluated (10% HCl for Bi and 10% citric acid for Cip and Cip-Bi). The sterile 96-well polystyrene microtitrate plates were prepared by dispersing 200 µL of appropriate dilution of the sample in a culture broth per well. Finally, 2 µL of a bacterial inoculum were added to the wells to obtain a density of 1.5 × 10^6^ CFU (colony-forming unit)/mL. After incubation (37 °C for 18 h), the panel was digitally analyzed at 600 nm using the hybrid multi-mode microplate reader (Synergy H4 BioTek laboratory instruments, Winooski, VE, USA) with the dedicated software system. The growth intensity in each well was compared with the controls: negative (sterile broth), positive (bacterial growth in broth without tested sample) and dye control (broth with tested sample without bacteria).

### 3.10. Statistical Analysis

The results are expressed as mean ± RSD (relative standard deviation). Microbiological testing results were evaluated statistically by analysis of variance (post-hoc, Tukey’s test) and statistical significance was set accordingly at the *p* < 0.05 level.

## 4. Conclusions

The threat of an increase in the number of bacteria resistant to classic antibiotics, including Cip, made us look for other solutions to inhibit the growth and multiplication of pathogens. It seems that such an alternative to a conventional Cip could be the Cip-Bi complex. Moreover, the combination of Cip with Bi may potentially reduce ciprofloxacin allergic reaction by reducing its absorption. The findings presented in this paper allow to conclude that the Cip-Bi-modified hydrogel dressing, obtained in an uncomplicated way, is a new wound dressing with potential wound healing properties in comparison to the unmodified dressings. We were able to incorporate the Cip-Bi complex into the dressing matrix successfully, which is confirmed by the obtained antimicrobial test results. The newly developed dressings show activity against tested reference and clinical Gram-positive and Gram-negative strains. Yet, in order to evaluate their potential application in practice, further research into this issue should focus on their safety and efficacy. It should be noted that there is insufficient evidence as yet to confirm the effect of antimicrobial dressings on wound healing in clinical practice. 

## Figures and Tables

**Table 1:** Evaluation of the ciprofloxacin-bismuth (Cip-Bi) loaded into the dressing matrix. Cip-Bi hydrogel solution before (solution A) and after (solution B) dressing modification.

Parameters	Solution A, mg/mL	Solution B, mg/mL	Volume of Solution B, mL *
Mean value, *n* = 6	1.1702	1.1864	0.6
Minimum/maximum	1.0831/1.2342	1.0627/1.3156	0.5/0.7
Confidence interval, ±95%	1.1129/1.2274	1.0823/1.2906	0.53/0,67
Standard deviation	0.0545	0.0992	0.07
Standard error	0.0223	0.0405	0.03
Relative standard deviation, %	4.66	8.36	11.8

* When the volume of solution A used to modify the dressing samples was 1 mL.

**Table 2:** Minimal inhibitory concentrations (MICs) and zones of inhibition determined for the solutions of ciprofloxacin (Cip), bismuth citrate (Bi) and ciprofloxacin-bismuth complex (Cip-Bi); mean ± relative standard deviation %, *n* = 5.

Bacteria	MIC Values (μg/mL)			Inhibition Zone Diameter (mm)		
	Bi	Cip	Cip-Bi	Bi	Cip	Cip-Bi
*S. aureus* ^ATCC 25923^	267	0.3125	0.2603	3.9 ± 5.1	25.2 ± 3.2	27.7 ± 5.1
*S. aureus* ^c.i.w.^	400	0.3122	0.4163	1.0 ± 14.5	21.7 ± 6.8	25.8 ± 2.0
*E. coli* ^ATCC 25992^	200	0.0098	0.0065	10.6 ± 4.4	28.7 ± 3.4	30.4 ± 7.7
*E. coli* ^c.i.w^	167	0.0781	0.0390	10.3 ± 10.4	24.8 ± 3.3	29.2 ± 2.8
*E. coli* ^c.i.u^	200	0.1560	0.1560	10.9 ± 7.3	26.1 ± 3.1	28.5 ± 1.6

Reference strains (ATCC, American Type Culture Collection), clinical isolates from infected wounds (c.i.w.) or urine (c.i.u.).

**Table 3:** Analysis of the effect of ciprofloxacin-bismuth (Cip-Bi)-modified dressing samples on the growth of the tested Gram-positive and Gram-negative bacteria (mean ± relative standard deviation %, *n* = 5).

Bacteria	Inhibition Zone Diameter (mm)						
	S1	S2/Br_2_	S3/I_2_	S4/Br_2_/Hydrogel	S5/I_2_/Hydrogel	S6/Br_2_/Cip-Bi Hydrogel	S7/I_2_/Cip-Bi Hydrogel
*S. aureus* ^ATCC 25923^	0	0	0	shade	10.2 ± 14.9	19.5 ± 2.6	22.2 ± 8.7
*S. aureus* ^c.i.w.^	0	0	4 ± 11	shade	13.7 ± 5.4	22.2 ± 6.1	25.3 ± 5.9
*E. coli* ^ATCC 25992^	0	0	0	shade	6.8 ± 8.4	34.8 ± 10.6	36.0 ± 2.6
*E. coli* ^c.i.w.^	0	shade	11 ± 6.1	shade	13.0 ± 6.1	30.9 ± 5.4	33.0 ± 6.0
*E. coli* ^c.i.u.^	0	shade	10 ± 8.9	12.4 ± 7.3	15.0 ± 5.3	26.0 ± 3.1	29.0 ± 2.7

(S1): Sample 1—untreated dressing; (S2): Sample 2—bromine-activated (Br_2_) dressing; (S3): Sample 3—iodine-activated (I_2_) dressing; (S4): Sample 4—bromine-activated (Br_2_), hydrogel-loaded dressing; (S5): Sample 5—iodine-activated (I_2_), hydrogel-loaded dressing; (S6): Sample 6—bromine-activated (Br_2_), Cip-Bi hydrogel-loaded dressing; (S7): Sample 7—iodine-activated (I_2_), Cip-Bi hydrogel-loaded dressing; clinical isolates from infected wounds (c.i.w.) or urine (c.i.u.).
